# Global Illness and Deaths Caused by Rotavirus Disease in Children

**DOI:** 10.3201/eid0905.020562

**Published:** 2003-05

**Authors:** Umesh D. Parashar, Erik G. Hummelman, Joseph S. Bresee, Mark A. Miller, Roger I. Glass

**Affiliations:** * Centers for Disease Control and Prevention, Atlanta, Georgia, USA; † Fogarty International Center, National Institutes of Health, Bethesda, Maryland, USA

**Keywords:** diarrhea, rotavirus, mortality, deaths, morbidity, hospitalizations, disease burden, research

## Abstract

To estimate the global illness and deaths caused by rotavirus disease, we reviewed studies published from 1986 to 2000 on deaths caused by diarrhea and on rotavirus infections in children. We assessed rotavirus-associated illness in three clinical settings (mild cases requiring home care alone, moderate cases requiring a clinic visit, and severe cases requiring hospitalization) and death rates in countries in different World Bank income groups. Each year, rotavirus causes approximately 111 million episodes of gastroenteritis requiring only home care, 25 million clinic visits, 2 million hospitalizations, and 352,000–592,000 deaths (median, 440,000 deaths) in children <5 years of age. By age 5, nearly every child will have an episode of rotavirus gastroenteritis, 1 in 5 will visit a clinic, 1 in 60 will be hospitalized, and approximately 1 in 293 will die. Children in the poorest countries account for 82% of rotavirus deaths. The tremendous incidence of rotavirus disease underscores the urgent need for interventions, such as vaccines, to prevent childhood deaths in developing nations.

In 1985, de Zoysa and Feachem published their landmark review of the global prevalence of rotavirus disease (*1*). Their analyses indicated that rotavirus accounted for 6% of diarrhea episodes and 20% of deaths caused by diarrhea in children <5 years of age in developing countries. The incidence of rotavirus disease was observed to be similar in both industrialized and developing countries, suggesting that adequate control may not be achieved by improvements in water supply, hygiene, and sanitation. Consequently, the development, trial, and widespread use of rotavirus vaccines were recommended to prevent severe and fatal rotavirus disease.

Since then, rapid progress has been made in developing and testing several rotavirus vaccine candidates (*2*,*3*). In August 1998, a live, attenuated rotavirus vaccine (Rotashield, Wyeth Laboratories, Marietta, PA) was licensed in the United States and recommended for routine immunization of U.S. infants. However, 9 months later, the use of Rotashield was suspended because reports suggested a possible association with intussusception (*4*). After this association was confirmed, the recommendation for use of Rotashield was withdrawn and the manufacturer stopped vaccine production.

Efforts are ongoing to develop other rotavirus vaccines, and several candidates are undergoing clinical testing (*3*). In addition to their safety and efficacy, the decision to implement these new rotavirus vaccines will be based on considerations of risk-benefit and cost-effectiveness. Updated estimates of rotavirus disease prevalence are a prerequisite to formulating such policy and carrying out economic analyses as well as advocacy for the next generation of rotavirus vaccines. Furthermore, each country that considers using a rotavirus vaccine may want to review the prevalence of rotavirus disease in their setting.

Since 1985, deaths from diarrheal diseases in children have declined substantially around the world, and a recent analysis suggested that deaths from rotavirus infections might also have been reduced during this period (*5*,*6*). Furthermore, scant information is available on the global extent of illness from rotavirus disease, particularly hospitalizations, which constitute a major component of total rotavirus illness and deaths in industrialized nations. To provide updated estimates of the global illness and death from rotavirus disease in children, we reviewed studies of childhood deaths from diarrhea and of rotavirus infections published from 1986 to 2000. We also present preliminary estimates of country-specific mortality rates from rotavirus disease as targets for further study and refinement through local definition of problems. These findings should help policy makers assess the magnitude of the problem of rotavirus disease in their own countries and set priorities for interventions to prevent this disease.

## Methods

### Selection of Studies

The studies selected for this analysis were identified from a computer search of the scientific literature published in English between 1986 and 2000. To find studies of childhood deaths from diarrhea, we conducted a search using the keywords “childhood mortality,” “deaths,” and “diarrhea.” We added references by reviewing the citations in these articles and by consulting with experts in the field. Because most studies of diarrhea deaths were conducted in countries with a low-income population, we supplemented these studies with published reports of vital registration data to analyze child death patterns in selected countries with middle- and high-income populations.

To identify studies of rotavirus disease, we conducted a search using the keyword “rotavirus” and cross-linked the articles with a second set of articles obtained from a secondary search using these keywords: incidence, prevalence, public health, death rate, mortality, surveillance, burden, suffering, distribution, area, location, and country. We also searched for permutations of these root words: epidemiol, monitor, and geograph. We then reviewed the resulting linked set of articles and narrowed it down to articles with content that was relevant to the goals of this study. We identified additional citations from references in these articles. Studies of rotavirus were included if they continued for at least 1 year, contained data on children <5 years of age, and reporting using an enzyme immunoassay (EIA) or similar reliable assay to detect rotavirus. A listing on the studies included in the analyses is available in Appendix A.

### Analysis of Data

#### Rotavirus-Associated Illness

To estimate the extent of illness from rotavirus in children in developing countries, we first multiplied the total population of infants (0–11 months) and children (12–59 months) in those countries by the estimated annual incidence of diarrhea in the respective age groups (*5*,*7*). On the basis of published estimates from a study in Chile (*8*), we then distributed these diarrhea episodes into three settings: mild cases only requiring care at home; moderate cases requiring care in an outpatient clinic; and severe cases requiring hospitalization. Next, on the basis of studies we reviewed, we calculated the median proportion of diarrhea episodes attributable to rotavirus in each of the three settings. Finally, we multiplied the total number of diarrhea episodes in each setting by the estimated proportion attributable to rotavirus to yield the number of rotavirus cases in each setting.

To estimate the number of hospitalizations for rotavirus among children in industrialized countries, we multiplied estimates of the total population of children <5 years of age with rotavirus-associated hospitalization rates derived from published studies. To calculate clinic visits and episodes of rotavirus disease, we evaluated studies documenting the frequency of these outcomes relative to hospitalizations and multiplied the calculated total number of rotavirus-associated hospitalizations by corresponding factors. The figures thus obtained were combined with estimates of rotavirus illness in children in developing countries to yield the global extent of illness from rotavirus disease.

### Rotavirus-Associated Deaths

To estimate the total number of child deaths from diarrhea, we plotted (for each country with available data) the fraction of deaths of children <5 years of age attributable to diarrhea against per capita gross national product (GNP). Countries were classified on the basis of GNP per capita into World Bank Income Groups (low [<U.S.$756], low-middle [U.S.$756–$2,995], high-middle [U.S. $2,996–$9,265], high [>U.S. $9,265]) (*9*). For each income group, we calculated the median proportion of deaths of children <5 years of age attributable to diarrhea. We then multiplied the median proportion for each income group by the total number of deaths of children <5 years of age for each country in that income group to yield country-specific estimates of the mortality rate from diarrhea. These country-specific estimates were added to calculate the global mortality rate from diarrhea.

To estimate the fraction of diarrhea deaths attributable to rotavirus, we plotted the proportion of rotavirus infection detected in children hospitalized for diarrhea that was, by virtue of the need for hospitalization, presumed to be severe. These figures were again plotted against per capita GNP for each country to yield median rotavirus detection rates for countries in the four World Bank income groups. Previously estimated diarrhea mortality rates for each country in an income group was multiplied by the median rotavirus detection rate for that income group to yield the estimated number of rotavirus deaths by country. These figures were added to yield the number of global deaths from rotavirus diarrhea. For each income strata and overall, the risk of death from rotavirus diarrhea by 5 years of age was calculated by dividing the total number of live births by the total number of deaths from rotavirus.

## Results

### Rotavirus Disease in Children in Developing Countries

#### Total Number of Diarrhea Episodes

An estimated 125 million infants 0–11 months of age and 450 million children 1–4 years of age reside in developing countries. A recent review of 27 prospective studies from 20 countries published from 1990 to 2000 estimated the incidence of diarrhea as 3.8 episodes per child per year for children <11 months of age and 2.1 episodes per child per year for children 1–4 years of age (*5*). Multiplying these age-specific incidence data with the population of children in each age group yielded an overall estimate of approximately 1.4 billion diarrhea episodes per year in children <5 years of age (Table 1). Of these, 475 million episodes are estimated to occur in <11-month-old infants and 945 million episodes in children 1–4 years of age.

**Table 1:** Estimates of the annual number of diarrhea episodes among children <5 years of age in developing countries, by age group and setting^a^

	Age group		
	<11 mo	1–4 y	Total (<4 y)
Total population (x1,000)	125,000	450,000	575,000
No. of diarrhea episodes per child per y^b^	3.8	2.1	NA
Total diarrhea episodes (x1,000)	475,000	945,000	1,420,000
No. of episodes at home (x1,000)	418,950 (88.2)	868,455 (91.9)	1,287,405
No. of episodes in outpatients (x1,000)	48,925 (10.3)	74,655 (7.9)	123,580
No. of case-patients hospitalized (x1,000)	7,125 (1.5)	1,890 (0.2)	9,015

^a^Figures in parenthesis are percentages of total diarrhea episodes (*7*).

^b^From reference (*5*).

### Distribution of Diarrhea Episodes by Setting

A study from Chile demonstrated that in <11-month-old infants, 88.2% of diarrhea episodes required only care at home, 10.3% required a clinic visit, and 1.5% required hospitalization (*8*). In 1- to 4-year-old children, 91.9% of diarrhea episodes required only care at home, 7.9% required a clinic visit, and only 0.2% required hospitalization. The proportion of all diarrhea episodes requiring hospitalization was similar in another study from Thailand (*10*). Therefore, we applied the estimates from the Chilean study to the previously calculated total number of diarrhea episodes in each age group and distributed them into episodes requiring only home care, clinic visit, or hospitalization (Table 1). Of the total of approximately 1.4 billion diarrhea episodes in children <5 years of age, we estimated that 1.29 billion require home care only, 124 million require a clinic visit, and 9 million require hospitalization.

### Number of Rotavirus Episodes in Each Setting

To estimate the number of diarrhea cases in each setting that are attributable to rotavirus, we applied proportions calculated from studies of rotavirus in children in developing countries. The review of 24 community-based studies, 13 clinic-based studies, and 72 hospital-based studies indicated that rotavirus accounted for a median of 8.1%, 18.8%, and 21.3% of diarrhea episodes in the three settings, respectively (Table 2). By multiplying these setting-specific proportions with the total number of diarrhea episodes in each setting, we calculated that rotavirus annually causes approximately 104 million episodes of diarrhea requiring home care, 23 million clinic visits, and 1.9 million hospitalizations.

**Table 2:** Estimates of the annual number of episodes of rotavirus diarrhea among children <5 years of age in developing countries, by setting

	Home	Outpatient	Inpatient
Annual no. of diarrhea episodes (x1,000)	1,287,405	123,580	9,015
Median % of episodes with rotavirus (IQR)^a^I	8.1 (4.0–12.2)	18.8 (15.0–22.0)	21.3 (17.2–28.8)
Total rotavirus episodes (range) (x1,000)	104,280 (51,496–157,063)	23,233 (18,537–27,188)	1,920 (1,551–2,596)

^a^IQR, interquartile range.

### Illness from Rotavirus Disease in Children in Industrialized Countries

#### Hospitalizations

Examination of rotavirus-specific annual hospitalization incidence from several industrialized countries demonstrated a median rate of 445 per 100,000 children (interquartile range, 283–715 per 100,000) (*11*–*20*) (Table 3). By multiplying these incidence estimates with the total population of 50,016,000 children <5 years of age in industrialized nations, we estimated that a total of 223,000 (range 142,000–358,000) rotavirus-associated hospitalizations occur in children in industrialized nations.

**Table 3:** Annual incidence of hospitalizations for rotavirus gastroenteritis in children <5 years of age in selected industrialized countries

Country (reference)	Y	Annual incidence/per 100,000 children	Cumulative incidence by 5 y of age
Spain (*11*)	1989–1995	250	1 in 80
Netherlands (*12*)	1998	270	1 in 74
United States (*13*)	1993–1995	274	1 in 73
Poland (*14*)	1996	310	1 in 65
Sweden (*15*)	1993–1996	370^a^	1 in 54
United Kingdom (*16*)	1993–1994	520	1 in 38
Finland (*17*)	1985–1995	610	1 in 33
Australia (*18*)	1993–1996	750	1 in 27
Hungary (*19*)	1993–1996	840^a^	1 in 24
Australia (*20*)	1991–1993	870	1 in 23

^a^Incidence for children <4 years of age.

### Clinic Visits

No reliable estimates of rotavirus-associated clinic visit rates are available for children in industrialized countries. However, studies have shown that for each child hospitalized with rotavirus diarrhea, approximately 5–10 children require a visit to a healthcare facility or physician’s office (*17*,*21*,*22*). Therefore, we multiplied the estimated 223,000 rotavirus hospitalizations by a factor of 8 (range 5–10) to obtain an estimated total of approximately 1,781,000 (range 708,000–3,576,000) clinic visits for rotavirus disease in children <5 years of age.

### Episodes Requiring Only Home Care

Studies have estimated that for each child requiring medical attention for rotavirus disease, an additional three to five children develop symptomatic disease requiring only home-care (*21*,*22*). Therefore, we multiplied the estimated 1,781,000 clinic visits by a factor of 4 (range 3–5) to estimate a total number of 7,122,000 (range 2,123,000–17,881,000) episodes of rotavirus gastroenteritis requiring only home care in children <5 years of age.

### Overall Illness from Rotavirus Gastroenteritis Worldwide

By adding the total prevalence of rotavirus illness in children in developing and industrialized nations, we estimated that each year rotavirus causes approximately 111 million episodes of gastroenteritis that require home care only, 25 million clinic visits, and 2 million hospitalizations in children <5 years of age worldwide (Table 4).

**Table 4:** Annual global illness incidence from rotavirus disease among children <5 years age, by setting

	No. (range) of episodes of rotavirus disease (x1,000)		
Setting	Developing countries	Industrialized countries	Total
Home	104,280 (51,496–157,063)	7,122 (2,123–17,881)	111,402 (53,619–174,946)
Outpatient	23,233 (18,537–27,188)	1,781 (708–3,576)	25,017 (19,245–30,764)
Inpatient	1,920 (1,551–2,596)	223 (142–358)	2,143 (1,693–2,954)

### Deaths from Rotavirus Disease in Children <5 Years of Age Worldwide

**Figure 1:**
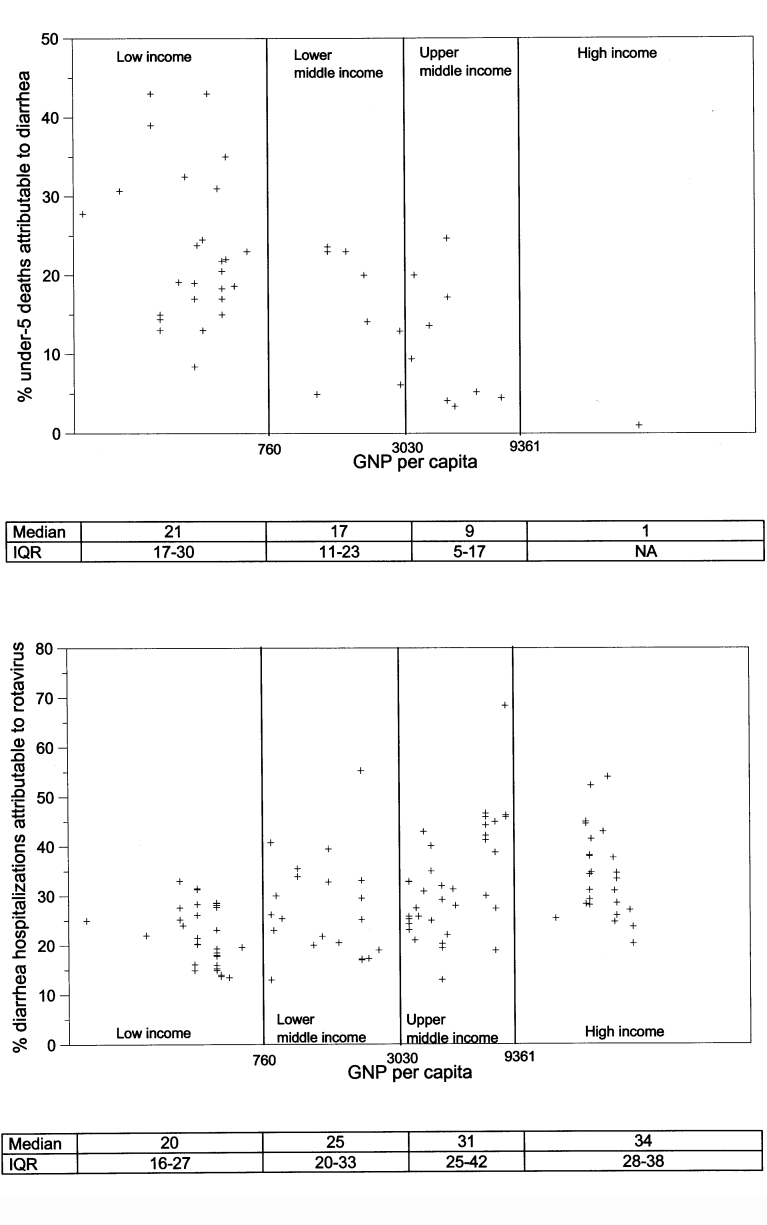
A. Percentage of deaths in children <5 years that are attributable to diarrhea for countries in different World Bank Income Groups, by gross national product (GNP) per capita of the country. B. Percentage of diarrhea hospitalization attributable to rotavirus for countries in different World Bank income groups, by GNP per capita of the country. IQR, interquartile range.

The proportion of deaths in children <5 years of age attributable to diarrhea demonstrated a declining trend with increasing income level (Figure 1A); the median proportion for low-income countries was 21%; for low-middle income countries, 17%; for high-middle income countries, 9%; and for high-income countries, 1%. We multiplied these income stratum-specific median estimates with the combined <5 mortality estimates for countries in each of the four income strata to yield an overall estimate of 2.1 million (range 1.7 million–3.0 million) diarrhea deaths per year (Table 5). Of the median 2.1 million diarrhea deaths, 85% (N=1,805,000) occurred in children from low-income countries.

**Table 5:** Global estimates of the annual number of diarrhea and rotavirus deaths among children <5 years of age, by income group

Income group	Total no. (x1,000)		Diarrhea deaths		Rotavirus deaths^b^		Risk of dying from rotavirus by age 5
	Births	Deaths	Median % (IQR^a^) of total deaths	Median no. (IQR) of deaths (x1,000)	Median % (IQR^a^) of diarrhea hospitalizations	Median no. (IQR) of deaths (x1,000)	
Low	70,447	8,595	21 (17–30)	1,805 (1,461–2,579)	20 (16–27)	361 (289–487)	1 in 205
Low middle	37,402	1,609	17 (11–23)	274 (177–370)	25 (20–33)	69 (55–90)	1 in 542
Upper middle	11,520	366	9 (5–17)	33 (18–62)	31 (25–42)	10 (8–14)	1 in 1,152
High	9,931	60	1	<1	34 (28–38)	<1	1 in 48,680
Total	129,300	10,630	NA	2,112 (1,657–3,012)	NA	440 (352–592)	1 in 293

^a^IQR, interquartile range.

^b^The estimated number and range of deaths from rotavirus are derived by multiplying the median and IQR of diarrhea hospitalizations attributable to rotavirus by the median number of deaths caused by diarrhea for each stratum.

The proportion of diarrhea hospitalizations attributable to rotavirus demonstrated an increasing trend with increasing income level (Figure 1B); the median for low-income countries was 20%; for low-middle income countries, 25%; for high-middle income countries, 31%; and for high-income countries, 34%. We multiplied these stratum-specific proportions with the median estimate of total diarrhea deaths for countries in each of the four income strata to yield an estimated 352,000–592,000 (median 440,000 deaths) per year from rotavirus. Of the median 440,000 deaths, 82% (N=361,000) occurred in children from low-income countries.

**Figure 2:**
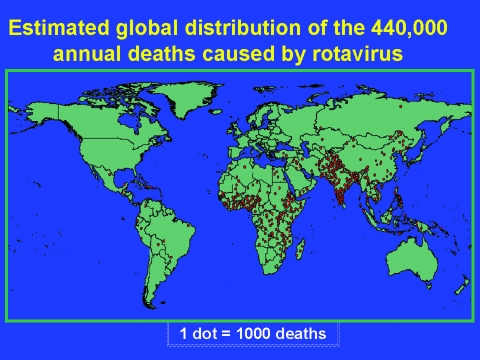
Estimated global distribution of 440,000 annual deaths in children caused by rotavirus diarrhea.

To obtain country-specific estimates of deaths from diarrhea and rotavirus disease, we first multiplied United Nations Children’s Fund estimates of total number of deaths of children <5 years of age for each country in each income stratum with the median proportion for that stratum of deaths in children <5 years of age attributable to diarrhea. The obtained country-specific estimates of diarrhea deaths were further multiplied by the median proportion for that stratum of diarrhea hospitalizations attributable to rotavirus. The results of these calculations are presented in the Appendix B and shown in Figure 2.

## Discussion

**Figure 3:**
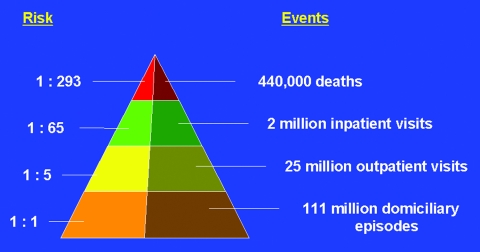
Estimated global prevalence of rotavirus disease.

The findings of this study demonstrate the tremendous amount of global illness and deaths caused by rotavirus disease. Each year, rotavirus causes an estimated 111 million episodes of diarrhea requiring only home-care, 25 million clinic visits, 2 million hospitalizations, and 352,000–592,000 deaths (median 440,000 deaths) in children <5 years of age. In other words, by 5 years of age, almost all children will have an episode of rotavirus gastroenteritis, 1 in 5 will require a clinic visit, 1 in 60 will require hospitalization, and approximately 1 in 293 will die (Figure 3). The incidence of rotavirus disease is similar in children in both developed and developing nations. However, children in developing nations die more frequently, possibly because of several factors, including poorer access to hydration therapy and a greater prevalence of malnutrition. An estimated 1,205 children die from rotavirus disease each day, and 82% of these deaths occur in children in the poorest countries.

In 1986, the Institute of Medicine (IOM) estimated, on the basis of published studies and field experience, that annually rotavirus causes approximately 110 million episodes of mild diarrhea, 10 million episodes of moderate to severe diarrhea, and 9 million episodes of severe diarrhea in children <5 years of age worldwide (*23*). Our estimate of the incidence of rotavirus gastroenteritis is similar to the IOM estimate and is consistent with a recent analysis demonstrating that overall diarrhea illness in children worldwide has not declined appreciably in the past two decades (*5*). However, our estimate of total hospitalizations from rotavirus disease is substantially lower than the IOM estimate. The difference might be explained, in part, by the relatively low hospitalization rate for diarrhea in the study in Chile (1.5% of all diarrhea episodes) used in our calculations (*8*). However, a study in a low-income urban community in Thailand showed a similar hospitalization rate (1% of all diarrhea episodes) among children with diarrhea (*10*), giving us added confidence in our estimates. Increased use of oral rehydration therapy and improvements in nutritional status are two factors that might explain a possible reduction in severe rotavirus cases without a concomitant decline in diarrhea incidence (*24*,*25*).

Our estimate of 352,000–592,000 deaths (median: 440,000 deaths) from rotavirus disease each year is similar to a recent estimate of 418,000–520,000 deaths proposed by Miller and McCann (*6*) but is substantially lower than the 1985 IOM estimate of 873,000 deaths. This decline in the rotavirus mortality rate parallels the decline in overall deaths from diarrhea in children in the past two decades, from an estimated 4.6 million deaths in 1982 (*26*) to our estimate of 2.1 million deaths in 2000. However, the patterns of diarrhea deaths reported in this study reflect the situation a decade ago, when most studies that we reviewed were conducted. Analyses of vital registration data from several countries have suggested that the proportion of deaths from diarrhea may have declined further in recent years (*27*). Other studies have noted marked discrepancies in the analysis of cause of death from vital registration data and prospective observational studies (*28*). Careful and detailed analyses are required to assess the current magnitude of the deaths from diarrhea in children, and the results will directly affect our estimates of deaths from rotavirus disease. For example, if our estimated proportion of severe diarrhea cases attributable to rotavirus is applied to the recent estimate of 2.5 million annual diarrhea deaths developed by Kosek et al. (*5*), we estimate 416,000–700,000 annual deaths (median:520,000 deaths) from rotavirus disease.

Another important factor that could affect our estimate of rotavirus deaths is the possibility that as the overall mortality rate from diarrhea has declined over the past two decades, the proportion of diarrhea deaths attributable to rotavirus may have increased, given that this pathogen is often transmitted from person to person and is difficult to control through improvements in hygiene and sanitation. This hypothesis is supported by data from Mexico, demonstrating that whereas deaths from diarrhea declined substantially from 1989 to 1995, the decline was less evident for winter seasonal deaths in children <2 years of age whose illness met the epidemiologic features of rotavirus diarrhea (*29*). In addition, some recent studies of rotavirus based on hospital surveillance in developing countries have demonstrated detection rates in excess of 50% (*30*,*31*). If this trend is confirmed as additional data become available from ongoing surveillance studies in several regions of the world, the estimates of rotavirus deaths reported in this article will have to be revised to reflect current mortality patterns.

This review, based on a compilation of studies varying in design, time, and place, has several inherent limitations that we attempted to address. Because of the marked seasonality of rotavirus disease and the variation in the sensitivity and specificity of diagnostic tests for rotavirus, we restricted this review to studies that lasted at least 1 year and used reliable assays for the detection of rotavirus. To account for known temporal changes in the magnitude and patterns of diarrhea-associated childhood deaths, we reviewed only studies published within the last 15 years and used the most recent available estimates of deaths in children <5 years to calculate estimates of deaths. Furthermore, because regional boundaries are primarily based on geographic and political considerations and do not necessarily reflect important determinants of health, we used indicators of socioeconomic status to stratify our analyses of mortality patterns.

Nevertheless, we could not adequately account for several factors that may have affected our findings. First, the studies we reviewed were conducted in selective populations that may not have been representative of the entire country. Second, most diarrhea mortality studies used verbal autopsies to determine the cause of death, which may affect our estimates because these methods have variable sensitivity and specificity and it is difficult, if not impossible, to assign a single cause of death for children who died with multiple conditions (*32*–*34*). Finally, because of a time lag between the conduct of studies and publication of their findings, our data likely do not reflect the most current trends of diarrhea and rotavirus disease prevalence and effects.

In 1998, the first rotavirus vaccine was licensed in the United States, offering an encouraging opportunity for the prevention of this disease. However, the vaccine was withdrawn within a year of licensure because it caused an estimated one case of intussusception for every 12,000 vaccinated infants. The lack of sufficient data on the efficacy of vaccine in developing countries as well as political and ethical considerations diminished prospects for its use in these settings. Our findings demonstrate that the next generation of rotavirus vaccines will have greatest impact in developing countries where the disease burden is greatest. Our estimates of rotavirus mortality rates for individual countries, although developed with relatively crude methods, compare favorably with those from more detailed analysis conducted in selected countries. For example, good concordance was noted between the previous figures and our estimates of rotavirus mortality for Bangladesh (14,850–27,000 vs. 12,449 deaths) (*35*), Peru (1,600 vs. 1,360 deaths) (*36*), and India (98,000 vs. 95,760 deaths) (*37*). The establishment of regional networks for rotavirus surveillance in sentinel hospitals will facilitate more timely and refined estimates of disease illness and death. These data, along with information on illness and costs of rotavirus infections, will assist policy makers in assessing the magnitude of the problem of rotavirus in their own setting and in setting priorities for interventions, such as the next generation of rotavirus vaccines, which may be available in the near future.

## Supplementary Material

Appendix A Studies of Rotavirus Diarrhea Included in this Analysis

Appendix B Country-specific estimates of deaths from diarrhea and rotavirus in children
