# Supplementary material for: Global Illness and Deaths Caused by Rotavirus Disease in Children
Source: Emerg Infect Dis. 2003 May;9(5):565–72. doi: 10.3201/eid0905.020562 (PMC2972763; doi:10.3201/eid0905.020562)
Supplement: Appendix A — Studies of Rotavirus Diarrhea Included in this Analysis [file 02-0562_appA-s1.pdf]

Appendix A. [online only] Studies of Rotavirus Diarrhea Included in this Analysis

1. Bhan MK, Bhandari N, Sazawal S, Clemens J, Raj P, Levine MM, et al. Descriptive epidemiology of persistent diarrhoea among young children in rural northern India. Bull World Health Organ 1989;67:281–8.
2. Raj P, Bhan MK, Prasad AK, Kumar R, Bhandari N, Jayashree S. Electrophoretic study of the genome of human rotavirus in rural Indian community. Indian J Med Res 1989;89:65–8.
3. Nath G, Shukla BN, Reddy DCS, Sanyal SC. A community study on the aetiology of childhood diarrhoea with special reference to *Campylobacter jejuni* in a semiurban slum of Varanasi, India. J Diarrhoeal Dis Res 1993;11:165–8.
4. Nath G, Singh SP, Sanyal SC. Childhood diarrhoea due to rotavirus in a community. Indian J Med Res 1992;95:259–62.
5. Yachha SK, Singh V, Kanwar SS, Mehta S. Epidemiology, subgroups and serotypes of rotavirus diarrhea in north Indian communities. Indian J Pediatr 1994;31:27–33.
6. Schorling JB, Wanke CA, Schorling SK, McAuliffe JF, deSouza A, Guerrant RL. A prospective study of persistent diarrhea among children in an urban Brazilian slum. Am J Epidemiol 1990;132:144–56.
7. Black RE, de Romana GL, Brown KH, Bravbo N, Bazalar OG, Kanashiro HC. Incidence and etiology of infantile diarrhea and major routes of transmission in Huascar, Peru. Am J Epidemiol 1989;129:785–99.
8. Cravioto A, Reyes RE, Ortega R, Fernandez G, Hernandez R, Lopez D. Prospective study of diarrhoeal disease in a cohort of rural Mexican children: incidence and isolated pathogens during the first two years of life. Epidemiol Infect 1988;101:123–34.
9. Cruz JR, Caceres P, Cano F, Flores J, Bartlett A, Torun B. Adenovirus types 40 and 41 and rotaviruses associated with diarrhea in children from Guatemala. J Clin Microbiol 1990;28:1780–4.
10. Linhares AC, Gabbay YB, Freitas RB, da Rosa ES, Mascarenhas JDP, Loureiro ECB. Longitudinal study of rotavirus infections among children from Belem, Brazil. Epidemiol Infect 1989;102:129–45.
11. Perez-Schael I, Garcia D, Gonzalez M, Gonzalez R, Daoud N, Perez M, et al. Prospective study of diarrheal diseases in Venezuelan children to evaluate the efficacy of rhesus rotavirus vaccine. J Med Virol 1990;30:219–29.
12. Espinoza F, Paniagua M, Hallander H, Svensson L, Strannegard O. Rotavirus infections in young Nicaraguan children. Pediatr Infect Dis J 1997;16:564–71.
13. Perez-Schael I. The impact of rotavirus disease in Venezuela. J Infect Dis 1996 Sep;174(Suppl 1):S19–21.
14. Zaki AM, DuPont HL, el Alamy MA, Arafat RR, Amin K, Awad MM, et al. The detection of enteropathogens in acute diarrhea in a family cohort population in rural Egypt. Am J Trop Med Hyg 1986;35:1013–22.
15. Naficy AB, Abu-Elyazeed R, Holmes JL, Rao MR, Savarino SJ, Kim Y, et al. Epidemiology of rotavirus diarrhea in Egyptian children and implications for disease control. Am J Epidemiol 1999;150:770–7.
16. Varavithya W, Vathanophas K, Bodhidatta L, Punyaratabandhu P, Sangchai R, Athipanyakom S, et al. Importance of salmonellae and *Campylobacter jejuni* in the etiology of diarrheal disease among children less than 5 years of age in a community in Bangkok, Thailand. J Clin Microbiol 1990;28:2507–10.
17. Khalil K, Lindblom GB, Mazhar K, Khan SR, Kajiser B. Early child health in Lahore, Pakistan: VIII. Microbiology. Acta Paediatr 1993;390:S87–94.
18. Yap KL, Yasmin AM, Wong YH, Ooi YE, Tan SC, Jegathesan M, et al. A one year community-based study on the incidence of diarrhoea and rotavirus infection in urban and suburban Malaysian children. Med J Malaysia 1992;47:303–8.
19. Coker AO, Dosunmu-Ogunbi O, Odugbemi T, Alabi SA, Macaulay SA. A study on the prevalence of rotavirus diarrhoeas in Ohazora local government area, Imo State, Nigeria, and the Lagos University Teaching Hospital, Lagos, Nigeria. East Afr Med J 1987;586–9.
20. Nakano T, Binka FN, Afari EA, Agbodaze D, Aryeetey ME, Mingle JAA, et al. Survey of enteropathogenic agents in children with and without diarrhoea in Ghana. J Trop Med Hyg 1990;93:408–12.
21. Oyejide CO, Fagbami AH. An epidemiological study of rotavirus diarrhoea in a cohort of Nigerian infants: II. Incidence of diarrhoea in the

first two years of life. Int J Epidemiol 1988;17:908–12.
22. Tswana SA, Jorgensen PH, Halliwell RW, Kapaata R, Moyo SR. The incidence of rotavirus infection in children from two selected study areas in Zimbabwe. Cent Afr J Med 1990;36:241–6.
23. Molbak K, Wested N, Hojlyng N, Scheutz F, Gottschau A, Aaby P, et al. The etiology of early childhood diarrhea: a community study from Guinea-Bissau. J Infect Dis 1994;169:581–7.
24. Punyaratabandhu P, Vathanophas K, Varavithya W, Sangchai R, Athipanyakom S, Echeverria P, et al. Childhood diarrhoea in a low-income urban community in Bangkok: incidence, clinical features, and child caretaker's behaviours. J Diarrhoeal Dis Res 1991;9:244–9.
25. Bhan MK, Raj P, Bhandari N, Svensson L, Stintzing G, Prasad AK, et al. Role of enteric adenoviruses and rotaviruses in mild and severe acute enteritis. Pediatr Infect Dis J 1988;7:320–3.
26. Broor S, Husain M, Chatterjee B, Chakraborty A, Seth P. Temporal variation in the distribution of rotavirus electropherotypes in Delhi, India. J Diarrhoeal Dis Res 1993;11:14–8.
27. Racz ML, Candeias JA, Trabulsi JR, Murahowski J. Diarrheal diseases in Brazil: clinical features of rotavirus-associated gastroenteritis in children. Eur J Epidemiol 1988;4:382–5.
28. Stewien KE, da Cunha LC, Alvim A de C, dos Reis Filho SA, Alvim MA, Brandao AA, et al. Rotavirus associated diarrhoea during infancy in the city of S. Luis (MA), Brazil: a two-year longitudinal study. Revista do Instituto de Medicina Tropical de Sao Paulo 1991;33:459–64.
29. Candeias JAN, Racz ML, Travulsi LR, Murahowsky J. Relative prevalence of rotavirus diarrhoea in children attending outpatient departments of hospitals and general practitioners in Sao Paulo, Brazil. J Diarrhoeal Dis Res 1989;7:24–7.
30. Perez-Schael I, Gonzalez R, Fernandez R, Alfonzo E, Inaty D, Boher Y, et al. Epidemiological features of rotavirus infection in Caracas, Venezuela: implications for rotavirus immunization programs. J Med Virol 1999;59:520–6.
31. Dutta SR, Khalfan SA, Baig BH, Philipose L, Fulayfil R. Epidemiology of rotavirus diarrhoea in children under five years in Bahrain. Int J Epidemiol 1990;19:722–7.
32. Pazzaglia G, Bourgeois AL, Araby I, Mikhail I, Podgore JK, Mourad A, et al. Campylobacter-associated diarrhoea in Egyptian infants: epidemiology and clinical manifestations of disease and high frequency of concomitant infections. J Diarrhoeal Dis Res 1993;11:6–13.
33. Herrmann JE, Blacklow NR, Perron-Henry DM, Clements E, Taylor DN, Echeverria P. Incidence of enteric adenoviruses among children in Thailand and the significance of these viruses in gastroenteritis. J Clin Microbiol 1988;26:1783–6.
34. Bingnan F, Unicomb L, Rahim Z, Nahar Banu N, Podder G, Clemens J, et al. Rotavirus-associated diarrhea in rural Bangladesh: two-year study of incidence and serotype distribution. J Clin Microbiol 1991;29:1359–63.
35. Phetsouvanh R, Midorikawa Y, Nakamura S. The seasonal variation in the microbial agents implicated in the etiology of diarrheal diseases among children in Lao People's Democratic Republic. Southeast Asian J Trop Med Public Health 1999;30:319–23.
36. Faruque ASG, Mahalanabis D, Islam A, Hoque SS, Hasnat A. Common diarrhea pathogens and the risk of dehydration in young children with acute watery diarrhea: a case-control study. Am J Trop Med Hyg 1993;49:93–100.
37. Armah GE, Mingle JA, Dodoo AK, Anyanful A, Antwi R, Commey J, et al. Seasonality of rotavirus infection in Ghana. Ann Trop Paediatr 1994;14:223–9.
38. Wu H, Taniguchi K, Urasawa T, Urasawa S. Serological and genomic characterization of human rotaviruses detected in China. J Med Virol 1998;55:168–76.
39. Fang ZY, Yang H, Zhang J, Li YF, Hou AC, Ma L, et al. Child rotavirus infection in association with acute gastroenteritis in two Chinese sentinel hospitals. Pediatr Int 2000;42:401–5.
40. Huilan S, Zhen LG, Mathan MM, Mathew MM, Olarte J, Espejo R, et al. Etiology of acute diarrhoea among children in developing countries: a multicentre study in five countries. Bull World Health Organ 1991;69:549–55.
41. Malik A, Rattan A, Malik MA, Shukla I. Rotavirus diarrhoea of infancy and childhood in a North Indian town—epidemiological aspects. J Trop Pediatr 1987;33:243–5.
42. Brown DW, Mathan MM, Mathew M, Martin R, Beards GM, Mathan VI. Rotavirus epidemiology in Vellore, south India: group, subgroup,

serotype, and electrophoretype. J Clin Microbiol 1988;6:2410–4.
43. Singh PB, Sreenivasan MA, Pavri KM. Viruses in acute gastroenteritis in children in Pune, India. Epidemiol Infect 1989,102:345–53.
44. Desai HS, Banker DD. Rotavirus infection among children in Bombay. Indian J Med Sci 1993;47:27–33.
45. Chakravarti A, Kumar S, Mittal SK, Broor S. Clinical and epidemiological features of acute gastroenteritis caused by human rotavirus subgroups. J Diarrhoeal Dis Res 1992;10:21–4.
46. Aggarwal P, Singh M, Guha DK. Prevalence of bacterial pathogens and rotavirus in hospitalised children with acute diarrhoea in Delhi, India. J Diarrhoeal Dis Res 1988;6:37–8.
47. Kelkar SD, Purohit SG, Simha KV. Prevalence of rotavirus diarrhoea among hospitalized children in Pune, India. Indian J Med Res 1999;109:131–5.
48. Ballal M, Jyothirlatha, Kotigadde S, Venkatesh A, Shivananda PG. Rotavirus and bacterial enteropathogens causing acute diarrhea. Ind J Pediatr 1992;59:203–7.
49. Chakravarti A, Broor S, Natarajan R, Setty VS, Mittal SK. Epidemiological and clinical characteristics of acute diarrhoea in children due to human rotavirus. J Trop Pediatr 1992;38:192–3.
50. Patwari AK, Srinivasan A, Diwan N, Aneja S, Anand VK, Peshin S. Rotavirus as an aetiological organism in acute watery diarrhoea in Delhi children: reappraisal of clinical and epidemiological characteristics. J Trop Pediatr 1994;40:214–8.
51. Husain M, Seth P, Broor S. Detection of group A rotavirus by reverse transcriptase and polymerase chain reaction in feces from children with acute gastroenteritis. Arch Virol 1995;140:1225–33.
52. Singh V, Broor S, Mehta S, Mehta SK. Molecular epidemiology of human rotavirus infections in Chandigarh (India). Indian J Med Res 1990;91:9–14.
53. Ghosh SK, Naik TN. Evidence for a new rotavirus subgroup in India. Epidemiol Infect 1989;102:523–30.
54. Aggarwal P, Srivastav VK, Singh M, Khanna KK. Rotavirus shown to be the main cause of acute childhood diarrhoea in a New Delhi Hospital with a high prevalence in winter. J Diarrhoeal Dis Res 1988;6:39–40.
55. Singh V, Broor S, Mehta S, Mehta SK. Clinical and epidemiological features of acute gastroenteritis associated with human rotavirus subgroups 1 and 2 in northern India. Indian J Gastroenterol 1989;8:23–5.
56. Mathew M, Mathan MM, Mani K, George R, Jebakumar K, Dharamsi R, et al. The relationship of microbial pathogens to acute infectious diarrhoea of childhood. J Trop Med Hyg 1991;94:253–60.
57. Dowe G, King SD, Maitland PB, Swaby-Ellis DE. Laboratory investigations on rotavirus in infantile gastroenteritis in Jamaica. Trans R Soc Trop Med Hyg 1988;82:155–9.
58. Stewien KE, Mos EN, Yanaguita RM, Jerez JA, Durigon EL, Harsi CM, et al. Viral, bacterial and parasitic pathogens associated with severe diarrhoea in the city of Sao Paulo, Brazil. J Diarrhoeal Dis Res 1993;11:148–52.
59. Cardoso D, Martins RM, Kitajima EW, Barbosa AJ, Camarota SC, Azevedo MS. Rotavirus and adenovirus in 0- to 5-year-old children hospitalized with or without gastroenteritis in Goiana, GO, Brazil. Revista do Instituto de Medicina Tropical de Sao Paulo 1992;34:433–9.
60. Teixeira JM, de Figueiredo RB, dos Santos HM, Ferreira MN, Camara GN. Epidemiology of rotavirus infections in the Federal District, Brazil. Revista Da Sociedade Brasileira de Medicina Tropical 1991;24:223–30.
61. Puerto FI, Polanco GG, Gonzalez MR, Zavala JE Jr, Ortega G. Role of rotavirus and enteric adenovirus in acute paediatric diarrhoea at an urban hospital in Mexico. Trans R Soc Trop Med Hyg 1989;83:396–8.
62. Suzuki H, Sato T, Kitaoka S, Tazawa F, Konno T, Amano Y, et al. Epidemiology of rotavirus in Guayaquil, Ecuador. Am J Trop Med Hyg 1986;35:372–5.
63. Urquidi V. Molecular eidemiology of human rotavirus infection in Coro, Venezuela. Acta Cientifica Venezolana 1989;40:33–9.
64. Perez-Schael I, Gonzalez R, Fernandez R, Alfonzo E, Inaty D, Boher Y, et al. Epidemiological features of rotavirus infection in Caracas, Venezuela: implications for rotavirus immunization programs. J Med Virol 1999;59:520–6.
65. Cama RI, Parashar UD, Taylor DN, Hickey T, Figueroa D, Ortega YR, et al. Enteropathogens and other factors associated with severe

disease in children with acute watery diarrhea in Lima, Peru. J Infect Dis 1999;179:1139–44.
66. Shukry S, Zaki AM, DuPont HL, Shoukry I, el Tagi M, Hamed Z. Detection of enteropathogens in fatal and potentially fatal diarrhea in Cairo, Egypt. J Clin Microbiol 1986;24:959–62.
67. el-Mougi M, Amer A, el-Abhar A, Hughes J, el-Shafie A. Epidemiological and clinical features of rotavirus associated acute infantile diarrhoea in Cairo, Egypt. J Trop Pediatr 1989;35:230–3.
68. Aithala G, Al Dhahry SH, Saha A, Elbualy MS. Epidemiological and clinical features of rotavirus gastroenteritis in Oman. J Trop Pediatr 1996;42:54–7.
69. Dagan R, Bar-David Y, Sarov B, Katz M, Kassis I, Greenberg D, et al. Rotavirus diarrhea in Jewish and Bedouin children in the Negev region of Israel: epidemiology, clinical aspects and possible role of malnutrition in severity of illness. Pediatr Infect Dis J 1990;9:314–21.
70. Adkins HJ, Escamilla J, Santiago L, Ranoa C, Echeverria P, Cross JH. Two-year survey of etiologic agents of diarrheal disease at San Lazaro Hospital, Manila, Republic of Philippines. J Clin Microbiol 1987;25:1143–7.
71. Marjoribanks HC, Croxson MC, Potoi N, Bellamy AR. Infantile gastroenteritis in Western Samoa. New Zealand Med J 1988;101:195–7.
72. Pipittajan P, Kasempimolporn S, Ikegami N, Akatani K, Wasi C, Sinarachatanant P. Molecular epidemiology of rotaviruses associated with pediatric diarrhea in Bangkok, Thailand. J Clin Microbiol 1991;29:617–24.
73. Hasegawa A, Mukoyama A, Suzuki H, Inouye S, Chearskul S, Thongkrajai P, et al. Rotavirus infection of Thai infants: antigen detection, RNA electrophoresis and virus cultivation. J Diarrhoeal Dis Res 1987;5:165–70.
74. Bern C, Unicomb L, Gentsch JR, Banul N, Yunus M, Sack RB, et al. Rotavirus diarrhea in Bangladeshi children: correlation of disease severity with serotypes. J Clin Microbiol 1992;30:3234–8.
75. San Pedro MC, Walz SE. A comprehensive survey of pediatric diarrhea at a private hospital in Metro Manila. Southeast Asian J Trop Med Public Health 1991;22:203–10.
76. Doan TN, Nguyen VC. Preliminary study on rotavirus diarrhoea in hospitalized children at Hanoi. J Diarrhoeal Dis Res 1986;4:81–2.
77. Howard P, Alexander ND, Atkinson A, Clegg AO, Gerega G, Javati A, et al. Bacterial, viral and parasitic aetiology of paediatric diarrhoea in the highlands of Papua New Guinea. J Trop Pediatr 2000;46:10–4.
78. Suwatano O. Acute diarrhea in under five-year-old children admitted to King Mongkut Prachomklao Hospital, Phetchaburi province. J Med Assoc Thai 1997;80:26–33.
79. Hoque SS, Faruque AS, Mahalanabis D, Hasnat A. Infectious agents causing acute watery diarrhoea in infants and young children in Bangladesh and their public health implications. J Trop Pediatr 1994;40:351–4.
80. Mubashir M, Khan A, Baqai R, Iqbal J, Ghafoor A, Zuberi S, et al. Causative agents of acute diarrhoea in the first 3 years of life: hospital-based study. J Gastroenterol Hepatol 1990;5:264–70.
81. Mendis L, de Silva D, Soysa P, Lamabadusuriya SP. Rotavirus infection in children hospitalised with diarrhoea in Sri Lanka. J Diarrhoeal Dis Res 1990;8:90–3.
82. Khan MM, Iqbal J, Ghafoor A, Burney MI. Aetiologic agents of diarrhoeal diseases in hospitalised children in Rawalpindi, Pakistan. J Diarrh Dis Res 1988;6:228–31.
83. Yap KL, Wong YH, Khor CM, Ooi YE. Rotavirus electropherotypes in Malaysian children. Can J Microbiol 1992;38:996–9.
84. Sirisanthana V, Leechanachai P, Poocharoen L. A clinical study of rotavirus diarrhea in Thai children. J Med Assoc Thai 1987;70:567–73.
85. Tabassum S, Shears P, Hart CA. Genomic characterization of rotavirus strains obtained from hospitalized children with diarrhoea in Bangladesh. J Med Virol 1994;43:50–6.
86. Albert MJ, Faruque AS, Faruque SM, Sack RB, Mahalanabis D. Case-control study of enteropathogens associated with childhood diarrhea in Dhaka, Bangladesh. J Clin Microbiol 1999;37:3458–64.
87. Nishio O, Matsui K, Oka T, Ushijima H, Mubina A, Dure-Samin A, et al. Rotavirus infection among infants with diarrhea in Pakistan. Pediatr Int 2000;42:425–7.
88. Maneekarn N, Ushijima H. Epidemiology of rotavirus infection in Thailand. Pediatr Int 2000;42:415–21.

89. Unicomb LE, Kilgore PE, Faruque SG, Hamadani JD, Fuchs GJ, Albert MJ, et al. Anticipating rotavirus vaccines: hospital-based surveillance for rotavirus diarrhea and estimates of disease burden in Bangladesh. Pediatr Infect Dis J 1997;16:947–51.
90. Teka T, Faruque AS, Fuchs GJ. Risk factors for deaths in under-age-five children attending a diarrhoea treatment centre. Acta Paediatr 1996;85:1070–5.
91. Mpabalwani M, Oshitani H, Kasolo F, Mizuta K, Luo N, Matsubayashi N, et al. Rotavirus gastro-enteritis in hospitalized children with acute diarrhoea in Zambia. Ann Trop Paediatr 1995;15:39–43.
92. Kakai R, Wamola IA, Bwayo JJ, Ndinya-Achola JO. Enteric pathogens in malnourished children with diarrhoea. East Afr Med J 1995;72:288–9.
93. Saidi SM, Iijima Y, Sang WK, Mwangudza AK, Oundo JO, Taga K, et al. Epidemiological study on infectious diarrheal diseases in children in a coastal rural area of Kenya. Microbiol Immunol 1997;41:773–8.
94. Gomwalk NE, Gosham LT, Umoh UJ. Rotavirus gastroenteritis in pediatric diarrhoea in Jos, Nigeria. J Trop Pediatr 1990;36:52–5.
95. Casalino M, Yusuf MW, Nicoletti M, Bazzicalupo P, Coppo A, Colonna B, et al. A two-year study of enteric infections associated with diarrhoeal diseases in children in urban Somalia. Trans R Soc Trop Med Hyg 1988;82:637–41.
96. Coker AO, Dosunmu-Ogunbi O, Odugbemi T, Alabi SA, Macaulay SA. A study on the prevalence of rotavirus diarrhoeas in Ohazora local government area, Imo State, Nigeria, and the Lagos University Teaching Hospital, Lagos, Nigeria. East Afr Med J 1987;586–9.
97. Tazi-Lakhsassi L, Garbarg-Chenon A, Nicolas JC, Soubhi H, Benbachir M, el Mdaghri N, et al. Epidemiological and clinical study and electrophoretyping survey of rotavirus acute diarrhoea in a children’s infectious disease unit in Casablanca, Morocco. Annales de l Institut Pasteur Virol 1988;139:205–15.
98. Gomwalk NE, Umoh UJ, Gosham LT, Ahmad AA. Influence of climatic factors on rotavirus infection among children with acute gastroenteritis in Zaria, northern Nigeria. J Trop Pediatr 1993;39:293–7.
99. Koulla-Shiro S, Loe C, Ekoe T. Prevalence of *Campylobacter enteritis* in children from Yaounde (Cameroon). Cent Fr J Med 1995;41:91–4.
100. Cisse MF, Ouangre RA, Gaye A, Boye CS, Sow AI, Mboup S, et al. Causes of infectious gastro-enteritis in children in Dakar. Presse Med 1989;18:1827–30.
101. Mefane C, Richard-Lenoble D, Gendrel D, Engonah E. Infantile diarrhea in Libreville (Gabon). Ecological studies. Arch Fr Pediatr 1986;43:813–6.
102. Tchambaz M, Messaoudi Z, Meziane O, Ammari H. Detection of rotavirus in the stools of infants aged 0–3 yr (study performed from July 1987 to May 1989). Arch Inst Pasteur Alger 1989;57:83–103.
103. Trabelsi A, Peenze I, Pager C, Jeddi M, Steele D. Distribution of rotavirus VP7 serotypes and VP4 genotypes circulating in Sousse, Tunisia, from 1995 to 1999: emergence of natural human reassortants. J Clin Microbiol 2000;38:3415–9.
104. Bos P, Mnisi YN, Steele AD. The molecular epidemiology of rotavirus infection in Ga-Rankuwa, southern Africa. Cent Afr J Med 1992;38:286–90.
105. Steele AD, Geyer A, Alexander JJ, Crewe-Brown HH, Fripp PJ. Enteropathogens isolated from children with gastro-enteritis at Ga-Rankuwa Hospital, South Africa. Ann Trop Paediatr 1988;8:262–7.
106. Griffiths FH, Steele AD, Alexander JJ. The molecular epidemiology of rotavirus-associated gastro-enteritis in the Transkei, Southern Africa. Ann Trop Pediatr 1992;12:259–64.
107. Steele AD, Alexander JJ. The relative frequency of subgroup I and II rotaviruses in black infants in South Africa. J Med Virol 1988;24:321–7.
108. Steele AD, Bos P, Alexander JJ. Clinical features of acute infantile gastroenteritis associated with human rotavirus subgroups I and II. J Clin Microbiol 1988;26:2647–9.
109. Visser LE, Cano Portero R, Gay NJ, Martinez Navarro JF. Impact of rotavirus disease in Spain: an estimate of hospital admissions due to rotavirus. Acta Paediatr 1999;88:S72–6.
110. deWit MAS, Koopmans MPG, van der Blig JF, van Duynhoven YTHP. Hospital admissions for rotavirus infection in the Netherlands. Clin

Infect Dis 2000;31:698–704.
111. Parashar UD, Holman RC, Clarke MJ, Bresee JS, Glass RI. Hospitalizations associated with rotavirus diarrhea in the United States, 1993 through 1995: surveillance based on the new ICD-9-CM rotavirus-specific diagnostic code. J Infect Dis 1998;177:13–7.
112. Mrukowicz JZ, Krobicka B, Duplaga M, Kowalska-Duplaga K, Domanski J, Szajewska H, et al. Epidemiology and impact of rotavirus diarrhoea in Poland. Acta Paediatr 999;88:53–60.
113. Johansen K, Bennet R, Bondesson K, Eriksson M, Hedlund KO, De Verdier Klingenberg K, et al. Incidence and estimates of the disease burden of rotavirus in Sweden. Acta Paediatr 1999;426:S20–3.
114. Ryan MJ, Ramsay M, Brown D, Gay NJ, Farrington CP, Wall PG. Hospital admissions attributable to rotavirus infection in England and Wales. J Infect Dis 1996;174(Suppl 1):S12–8.
115. Vesikari T, Rautanen T, Bonsdorff CHV. Rotavirus gastroenteritis in Finland: burden of disease and epidemiological features. Acta Pediatr 1999;426:S24–30.
116. Carlin JB, Chondros P, Masendycz P, Bugg H, Bishop RF, Barnes GL. Rotavirus infection and rates of hospitalisation for acute gastroenteritis in young children in Australia, 1993-1996. Med J Aust 1998;169:252–6.
117. Szucs G, Uj M, Mihaly I, Deak J. Burden of human rotavirus-associated hospitalizations in three geographic regions of Hungary. Acta Paediatr 1999;426:S61–5.
118. Ferson MJ. Hospitalizations for rotavirus gastroenteritis among children under five years of age in New South Wales. Medical Journal of Australia 1996;164:273–6.
119. Ford-Jones EL, Wang E, Petric M, Corey P, Moineddin R, Fearon M, et al. Rotavirus-associated diarrhea in outpatient settings and child care centers. Arch Pediatr Adolesc Med 2000;154:586–93.
120. Rodriguez WJ, Kim HW, Brandt CD, Schwartz RH, Gardner MK, Jeffries B, et al. Longitudinal study of rotavirus infection and gastroenteritis in families served by a pediatric medical practice: clinical and epidemiologic observations. Pediatr Infect Dis J 1987;6:170–6.
121. Donelli G, Ruggeri FM, Tinari A, Marziano ML, Caione D, Concato C, et al. A three-year diagnostic and epidemiological study on viral infantile diarrhea in Rome. Epidem Infect 1988;100:311–20.
122. Ruggeri FM, Marziano ML, Tinari A, Salvatori E, Donelli G. Four-year study of rotavirus electropherotypes from cases of infantile diarrhea in Rome. J Clin Microbiol 1989;27:1522–6.
123. Arista S, Giovannelli L, Pistoia D, Cascio A, Parea M, Gerna G. Electropherotypes, subgroups, and serotypes of human rotavirus strains causing gastroenteritis in infants and young children in Palermo, Italy, from 1985 to 1989. Res Virol 1990;141:435–48.
124. Barnes GL, Uren E, Stevens KB, Bishop RF. Etiology of acute gastroenteritis in hospitalized children in Melbourne, Australia, from April 1980 to March 1993. J Clin Microbiol 1998;36:133–8.
125. Caprioli A, Pezzella C, Morelli R, Giammanco A, Arista S, Crotti D, et al. Enteropathogens associated with childhood diarrhea in Italy. Pediatr Infect Dis J 1996;15:876–83.
126. Superti F, Diamanti E, Giovannangeli S, Dobi V, Xhelili L, Donelli G. Electropherotypes of rotavirus strains causing gastroenteritis in infants and young children in Tirana, Albania, from 1988 to 1991. Acta Virol 1995;39:257–61.
127. Muller FM, Onder G, Kamin W, Gutjahr P, Schmitt HJ. Diarrhea in 1,337 children of the Mainz University Clinic: Importance of *Salmonella* and rotaviruses. Klinische Padiatrie 1993;205:9–13.
128. Gosciniak G, Sobieszczanska B, Grzybek-Hryncewicz K. Rotavirus diarrhea in children hospitalized in Wroclaw clinics. Przeglad Lekarski 1990;47:682–5.
129. Pazdiora P, Taborska J, Mlada L, Kobesova A. Clinical and epidemiologic findings from a study of rotavirus infections in hospitalized children. Ceskoslovenska Epidemiologie Mikrobiologie Imunologie 1990;39:149–54.
130. Sadurska E. Studies on the role of rotaviruses in the etiology of acute diarrhea in infants. Pediatria Polska 1989;64:2–8.
131. Begue RE, Neill MA, Papa EF, Dennehy PH. A prospective study of shiga-like toxin-associated diarrhea in a pediatric population. J Pediatr Gastroenterol Nutr 1994;19:164–9.

132. Donelli G, Superti F, Tinari A, Marziano ML, Caione D, Concato C, et al. Viral childhood diarrhoea in Rome: a diagnostic and epidemiological study. Microbiologica 1993;16:215–26.
133. Grimwood K, Carzino R, Barnes GL, Bishop RF. Patients with enteric adenovirus gastroenteritis admitted to an Australian pediatric teaching hospital from 1981 to 1992. J Clin Microbiol 1995;33:131–6.
134. Sack RB, Santosham M, Reid R, Black R, Croll J, Yolken R, et al. Diarrhoeal diseases in the White Mountain Apaches: clinical studies. J Diarrhoeal Dis Res 1995;13:12–7.
135. Berner R, Schumacher RF, Hameister S, Forster J. Occurrence and impact of community-acquired and nosocomial rotavirus infections—a hospital-based study over 10 y. Acta Paediatr Suppl 1999;88:48–52.
136. Ginevskaya VA, Amitina NN, Eremeeva TP, Shirman GA, Priimagi LS, Drozdov SG. Electropherotypes and serotypes of human rotavirus in Estonia in 1989–1992. Arch Virol 1994;137:199–207.
137. Vizzi E, Cascio A, Arista S. Caratterizzazione antigenica di rotavirus umani riscontrati a Palermo negli anni 1989-92. Igiene Moderna 1994;102:547–55.
138. Gonzalez FS, Sordo ME, Rowensztein G, Sabbag L, Roussos A, De Petre E, et al. Rotavirus diarrhea. Impact in a pediatric hospital of Buenos Aires. Medicina (B Aires) 1999;59:321–6.
139. Uriarte S, Gomez J, Scolaro L. Rotavirus infection in children seen at a greater Buenos Aires hospital. Rev Argent Microbiol 1999;31:87–9.
140. Dutta SR, Khalfan SA, Baig BH, Philipose L, Fulayfil R. Epidemiology of rotavirus diarrhoea in children under five years in Bahrain. Int J Epidemiol 1990;19:722–7.
141. Milaat WA, el Assouli SM. Epidemiology of diarrhoea in two major cities in Saudi Arabia. J Communicable Dis 1995;27:84–91.
142. Mohammed KA, el Assouli SM, Banjar ZM. Human rotavirus subgroups and serotypes in children with acute gastroenteritis in Saudi Arabia from 1988 to 1992. J Med Virol 1994;44:237–42.
143. el Assouli SM, Banjar ZM, Mohammed KA, Zamakhchari FT. Rotavirus infection in children in Saudi Arabia. Am J Trop Med Hyg 1992;46:272–7.
144. Sethi SK, Khuffash F. Bacterial and viral causes of acute diarrhoea in children in Kuwait. J Diarrhoeal Dis Res 1989;7:85–8.
145. Huq MI, Rahman AS, Al-Sadiq A, Al-Shahri A, Alim AR. Rotavirus as an important cause of diarrhoea in a hospital for children in Dammam, Saudi Arabia. Ann Trop Paediatr 1987;7:173–6.
146. al-Bwardy MA, Ramia S, al-Frayh AR, Chagla AH, al-Omair AA, el-Hazmi MA, et al. Bacterial, parasitic and viral enteropathogens associated with diarrhoea in Saudi children. Ann Trop Paediatr 1988;8:26–30.
147. Khuffash FA, Sethi SK, Shaltout AA. Acute gastroenteritis: clinical features according to etiologic agents. Clin Pediatr (Phila) 1988;27:365–8.
148. el Assouli SM, Mohammed KA, Banjar ZM. Human rotavirus genomic RNA electropherotypes in Jeddah, Saudi Arabia from 1988 to 1992. Ann Trop Paediatr 1995;15:45–53.
149. el Assouli SM, Banjar ZM, Mohammed KA, Milaat WA, el Assouli MZ. Genetic and antigenic analysis of human rotavirus prevalent in Al-Taif, Saudi Arabia. J Trop Pediatr 1996;42:211–9.
150. Sethi S, Khuffash FA, Al-Nakib W. Microbial etiology of acute gastroenteritis in hospitalized children in Kuwait. Pediatr Infect Dis J 1989;8:593–7.
151. Kim KH, Suh IS, Kim JM, Kim CW, Cho YJ. Etiology of childhood diarrhea in Korea. J Clin Microbiol 1989;27:1192–6.
152. Tam JS, Kum WW, Lam B, Yeung CY, Ng MH. Molecular epidemiology of human rotavirus infection in children in Hong Kong. J Clin Microbiol 1986;23:660–4.
153. Chan PK, Tam JS, Nelson EA, Fung KS, Adeyemi-Doro FA, Fok TF, et al. Rotavirus infection in Hong Kong: epidemiology and estimates of disease burden. Epidemiol Infect 1998;120:321–5.
154. Biswas R, Lyon DJ, Nelson EA, Lau D, Lewindon PJ. Aetiology of acute diarrhoea in hospitalized children in Hong Kong. Trop Med Int Health 1996;1:679–83.

155. Kim KH, Yang JM, Joo SI, Cho YG, Glass RI, Cho YJ. Importance of rotavirus and adenovirus types 40 and 41 in acute gastroenteritis in Korean children. J Clin Microbiol 1990;28:2279–84.
156. Vijayan V, Quak SH, Wong HB. Incidence, clinical features and epidemiology of rotavirus gastro-enteritis in hospitalized children. Ann Trop Paediatr 1990;10:179–83.
157. Ling JM, Cheng AF. Infectious diarrhoea in Hong Kong. J Trop Med Hyg 1993;96:107–12.
158. Seo JK, Sim JG. Overview of rotavirus infections in Korea. Pediatr Int 2000;42:406–10.
159. Steele AD, Alexander JJ, Hay IT. Rotavirus-associated gastroenteritis in black infants in South Africa. J Clin Microbiol 1986;23:992–4.
160. Bos P, Mnisi YN, Steele AD. The molecular epidemiology of rotavirus infection in Ga-Rankuwa, southern Africa. Cent Afr J Med 1992;38:286–90.
161. Steele AD, Geyer A, Alexander JJ, Crewe-Brown HH, Fripp PJ. Enteropathogens isolated from children with gastro-enteritis at Ga-Rankuwa Hospital, South Africa. Ann Trop Paediatr 1988;8:262–7.
162. Griffiths FH, Steele AD, Alexander JJ. The molecular epidemiology of rotavirus-associated gastro-enteritis in the Transkei, South Afr Ann Trop Pediatr 1992;12:259–64.
163. Steele AD, Alexander JJ. The relative frequency of subgroup I and II rotaviruses in black infants in South Africa. J Med Virol 1988;24:321–7.
164. Steele AD, Bos P, Alexander JJ. Clinical features of acute infantile gastroenteritis associated with human rotavirus subgroups I and II. J Clin Microbiol 1988;26:2647–9.
