# Supplementary material for: Global Illness and Deaths Caused by Rotavirus Disease in Children
Source: Emerg Infect Dis. 2003 May;9(5):565–72. doi: 10.3201/eid0905.020562 (PMC2972763; doi:10.3201/eid0905.020562)
Supplement: Appendix B — Country-specific estimates of deaths from diarrhea and rotavirus in children [file 02-0562_appB-s2.pdf]

Appendix B [online only]

Table. Country-specific estimates of deaths from diarrhea and rotavirus in children.

Country	GNP per capita (US $)[a]	Deaths of children <5 y (x1,000)[b]	Estimated deaths from diarrhea[c]	Estimated deaths from rotavirus[d]
Afghanistan	250	293	61,530	12,306
Albania	870	2	340	85
Algeria	1,550	36	6,120	1,530
Angola	220	176	36,960	7,392
Antigua and Barbuda	8,520	0	–	–
Argentina	7,600	16	1,440	446
Armenia	490	1	210	42
Australia	20,050	1	10	3
Austria	25,970	0	–	–
Azerbaijan	550	5	1,050	210
Bahamas	12,400	0	–	–
Bahrain	7,640	0	–	–
Bangladesh	370	312	65,520	13,104
Barbados	6,610	0	–	–
Belarus	2,630	3	510	128
Belgium	24,510	1	10	3
Belize	2,730	0	–	–
Benin	380	38	7,980	1,596
Bhutan	510	8	1,680	336
Bolivia	1,010	22	3,740	935
Bosnia and Herzegovina	b	1	170	43
Botswana	3,240	3	270	84
Brazil	4,420	134	12,060	3,739
Brunei Darussalam	24,630	0	–	–
Bulgaria	1,380	1	170	43
Burkina Faso	240	105	22,050	4,410
Burundi	120	48	10,080	2,016
Cambodia	260	44	9,240	1,848
Cameroon	580	88	18,480	3,696
Canada	19,320	2	20	7
Cape Verde	1,330	1	170	43
Central African Republic	290	23	4,830	966
Chad	200	64	13,440	2,688
Chile	4,740	3	270	84
China	780	813	138,210	34,553
Colombia	2,250	31	5,270	1,318
Comoros	350	2	420	84
Congo	670	13	2,730	546
Congo, Dem. Republic	110	475	99,750	19,950
Costa Rica	2,740	1	170	43

Côte d'Ivoire	710	92	19,320	3,864
Croatia	4,580	0	–	–
Cuba	1,170	1	170	43
Cyprus	11,960	0	–	–
Czech Republic	5,060	0	–	–
Denmark	32,030	0	–	–
Djibouti	790	3	510	128
Dominica	3,170	0	–	–
Dominican Republic	1,910	10	1,700	425
Ecuador	1,310	11	1,870	468
Egypt	1,400	89	15,130	3,783
El Salvador	1,900	7	1,190	298
Equatorial Guinea	1,170	3	510	128
Eritrea	200	16	3,360	672
Estonia	3,480	0	–	–
Ethiopia	100	475	99,750	19,950
Fiji	2,210	0	–	–
Finland	23,780	0	–	–
France	23,480	4	40	14
Gabon	3,350	6	540	167
Gambia	340	4	840	168
Georgia	620	2	420	84
Germany	25,350	4	40	14
Ghana	390	73	15,330	3,066
Greece	11,770	1	10	3
Grenada	3,450	0	–	–
Guatemala	1,660	24	4,080	1,020
Guinea	510	56	11,760	2,352
Guinea-Bissau	160	10	2,100	420
Guyana	760	1	170	43
Haiti	460	33	6,930	1,386
Honduras	760	9	1,530	383
Hungary	4,650	1	90	28
Iceland	29,280	0	–	–
India	450	2,400	504,000	100,800
Indonesia	580	240	50,400	10,080
Iran	1,760	64	10,880	2,720
Iraq	2,170	103	17,510	4,378
Ireland	19,160	0	–	–
Israel	17,450	1	10	3
Italy	19,710	3	30	10
Jamaica	2,330	1	170	43
Japan	32,230	5	50	17
Jordan	1,500	8	1,360	340
Kazakhstan	1,230	12	2,040	510
Kenya	360	117	24,570	4,914
Kiribati	910	0	–	–
Korea, Dem. People’s Rep.	a	14	2,940	588

Korea, Republic of	8,490	3	270	84
Kuwait	19,020	0	–	–
Kyrgyzstan	300	8	1,680	336
Lao People’s Dem. Republic	280	23	4,830	966
Latvia	2,470	0	–	–
Lebanon	3,700	2	180	56
Lesotho	550	10	2,100	420
Liberia	490	30	6,300	1,260
Libya	5,540	4	360	112
Lithuania	2,620	1	170	43
Luxembourg	44,640	0	–	–
Madagascar	250	94	19,740	3,948
Malawi	190	105	22,050	4,410
Malaysia	3,400	5	450	140
Maldives	1,160	1	170	43
Mali	240	119	24,990	4,998
Malta	9,210	0	–	–
Marshall Islands	1,560	0	–	–
Mauritania	380	19	3,990	798
Mauritius	3,590	0	–	–
Mexico	4,400	77	6,930	2,148
Micronesia, Fed. States of	1,810	0	–	–
Moldova, Republic of	370	2	420	84
Mongolia	350	5	1,050	210
Morocco	1,200	37	6,290	1,573
Mozambique	230	168	35,280	7,056
Myanmar	220	106	22,260	4,452
Namibia	1,890	4	680	170
Nepal	220	82	17,220	3,444
Netherlands	24,320	1	10	3
New Zealand	13,780	0	–	–
Nicaragua	430	8	1,680	336
Niger	190	137	28,770	5,754
Nigeria	310	781	164,010	32,802
Norway	32,880	0	–	–
Oman	4,940	1	90	28
Pakistan	470	599	125,790	25,158
Panama	3,070	2	180	56
Papua New Guinea	800	17	2,890	723
Paraguay	1,580	5	850	213
Peru	2,390	32	5,440	1,360
Philippines	1,020	87	14,790	3,698
Poland	3,960	4	360	112
Portugal	10,600	1	10	3
Qatar	12,000	0	–	–
Romania	1,520	5	850	213
Russian Federation	2,270	32	5,440	1,360
Rwanda	250	53	11,130	2,226

Saint Kitts and Nevis	6,420	0	–	–
Saint Lucia	3,770	0	–	–
Saint Vincent/Grenadines	2,700	0	–	–
Samoa	1,060	0	–	–
Sao Tome and Principe	270	0	–	–
Saudi Arabia	6,910	17	1,530	474
Senegal	510	43	9,030	1,806
Seychelles	6,540	0	–	–
Sierra Leone	130	68	14,280	2,856
Singapore	29,610	0	–	–
Slovakia	3,590	1	90	28
Slovenia	9,890	0	–	–
Solomon Islands	750	0	–	–
Somalia	120	106	22,260	4,452
South Africa	3,160	73	6,570	2,037
Spain	14,000	2	20	7
Sri Lanka	820	6	1,020	255
Sudan	330	103	21,630	4,326
Suriname	1,660	0	–	–
Swaziland	1,360	3	510	128
Sweden	25,040	0	–	–
Switzerland	38,350	0	–	–
Syria	970	14	2,380	595
Tajikistan	290	14	2,940	588
Tanzania	240	188	39,480	7,896
TFYR Macedonia	1,690	1	170	43
Thailand	1,960	30	5,100	1,275
Togo	320	26	5,460	1,092
Tonga	1,720	0	–	–
Trinidad and Tobago	4,390	0	–	–
Tunisia	2,100	6	1,020	255
Turkey	2,900	68	11,560	2,890
Turkmenistan	660	9	1,890	378
Uganda	320	142	29,820	5,964
Ukraine	750	10	2,100	420
United Arab Emirates	17,870	0	–	–
United Kingdom	22,640	4	40	14
United States	30,600	30	300	102
Uruguay	5,900	1	90	28
Uzbekistan	720	38	7,980	1,596
Vanuatu	1,170	0	–	–
Venezuela	3,670	13	1,170	363
Viet Nam	370	66	13,860	2,772
Yemen	350	98	20,580	4,116
Yugoslavia	b	3	510	128
Zambia	320	76	15,960	3,192
Zimbabwe	520	32	6,720	1,344
**Total**		**10,630**	**2,112,020**	**439,788**

[a]GNP, gross national product.

[b]Figures for total deaths in children <5 y of age are rounded to the nearest thousand; consequently, estimates of diarrhea and rotavirus deaths are not calculated for countries with fewer than 1,000 child deaths.

[c]Deaths from diarrhea were derived by multiplying total deaths in children <5 y of age by the estimated proportion of deaths attributable to diarrhea, based on GNP per capita, as follows: GNP < US$756, 21%; GNP = US$756–$2,995, 17%; GNP = US$2,995–9,625, 9%; GNP > US$9,625, 1%.

[d]Deaths from rotavirus were derived by multiplying total diarrhea deaths in children <5 y of age by the estimated proportion of diarrhea deaths attributable to rotavirus, based on GNP per capita, as follows: GNP < US$756, 20%; GNP = US$756–$2,995, 25%; GNP = US$2,995–9,625, 31%; GNP > US$9,625, 34%.
